# Alignment and contribution of lean management practices to strategic objectives in a healthcare context: a qualitative study in a university medical centre

**DOI:** 10.1136/bmjopen-2025-099758

**Published:** 2025-12-15

**Authors:** Anouk Zuidland, Oskar Roemeling, Kjeld H Aij, Lihui Pu, Femke Vijn

**Affiliations:** 1 University of Groningen Faculty of Economics and Business, Groningen, Netherlands; 2 Department Dijkzigt, Erasmus MC, Rotterdam, Netherlands; 3 Department Internal Medicine, Section Nursing Science, Rotterdam, Erasmus MC University Medical Center Rotterdam, Rotterdam, Zuid-Holland, Netherlands

**Keywords:** QUALITATIVE RESEARCH, Quality Improvement, Change management, HEALTH SERVICES ADMINISTRATION & MANAGEMENT

## Abstract

**Abstract:**

**Objectives:**

This study investigates how lean management (LM) practices align with and contribute to strategic objectives in healthcare using the congruence model as a conceptual framework.

**Design:**

This study employed a qualitative research design, integrating both inductive and deductive approaches. Data were collected from multiple hospital departments using primary and secondary sources. Primary data included semi-structured interviews, guided by a standardised interview protocol. Secondary data consisted of reports on courses of action, annual reports, policy documents and findings from exploratory meetings. The analysis involved iterative cycles of open coding, axial coding and selective coding to systematically identify and refine themes, supporting thematic analysis.

**Setting:**

Six departments: Kidney and vascular surgery, Care core movement, Clinical department, Systemic diseases, Oncology, Care core Hepato-Pancreato-Biliary disease (HPB), which were part of one business unit within a tertiary care institution, a University Medical Centre in the Netherlands.

**Participants:**

22 respondents aged 18 years or older were purposefully selected based on their organisational roles and LM experience. Respondents had to be involved in LM practices, which meant they had to understand LM and have passed at least one LM training programme, or they should be enrolled in a training course during the research.

**Results:**

Interviewees identified several barriers to LM implementation, including insufficient management support for a culture of continuous improvement, limited time for LM projects, inadequate recognition from management and poor communication of strategic objectives. Despite positive individual experiences with LM projects, their contribution to strategic objectives was deemed limited due to these barriers. Specific challenges included an experienced lack of leadership commitment, inadequate follow-up on projects, insufficient resource allocation, limited access to training and leaders failing to acknowledge employee value. Facilitators of success were fostering a culture of continuous improvement, committed managers and providing training.

**Conclusions:**

LM practices have the potential to contribute to strategic objectives in healthcare organisations by reducing waste, improving patient care quality and optimising processes. However, their impact is constrained by organisational barriers and misalignments. Challenges such as insufficient resources, poor project follow-up, lack of management support and the absence of a continuous improvement culture minimise LM’s effectiveness.

**STRENGTHS AND LIMITATIONS OF THIS STUDY**

- The research site has a lot of lean management experience.
- The study has a comprehensive and varied qualitative data set.
- A conceptual framework rooted in systems thinking provided a conceptual lens.
- The qualitative nature of this study might limit generalisability of the findings.
- Qualitative research is inherently interpretive, and researcher’s bias might influence data collection and analysis.

## Introduction

 Healthcare organisations are facing unpredictable challenges such as improving patient outcomes, reducing healthcare costs and providing high quality patient care.^1^ The quality that healthcare organisations deliver to patients can decrease due to internal inefficiencies, such as poor patient flow and inadequate resource utilisation.^2 3^ This can bring down staff and patient satisfaction, which in turn could create additional costs for healthcare organisations.^4^ To cope with these challenges, healthcare organisations are searching for and adopting new management approaches.

One such approach for improving efficiency and reducing waste is lean management (LM). LM is a systematic approach to continuous improvement that focuses on eliminating waste, reducing costs and improving quality,^5^ and has its roots in the Toyota Production System (TPS).^6^ LM has the potential to reduce costs while increasing efficiency and has become popular in many healthcare organisations.^1 7 8^

Multiple studies have focused on the application of LM in healthcare, highlighting the relevance of management approaches, including LM, for improving patient care.^9^,^11^ Earlier research show cultural and practical barriers to embracing LM principles in hospitals.^12^ In addition, studies focused on the barriers, challenges and success factors of LM in healthcare organisations.^13^,^15^ In short, there is a broad set of LM-oriented studies available that cover a variety of topics. However, how LM practices align with the broader organisational strategy is not that clear.

The research of D’Andreamatteo *et al* states that even though LM results appear to be promising, findings so far do not allow us to draw a final conclusion on its positive impacts or challenges when introduced in the healthcare sector.^8^ With a specific focus on the alignment between LM and strategic goals in healthcare, this research contributes to increasing knowledge, identifying both positive impacts and challenges, and providing practical insights that can enrich the research field and practice of LM in healthcare. Furthermore, this research extends the study of Tlapa *et al*,^1^ which focused on specific outcomes like reduced wait times and length of stay.

A lack of alignment between LM projects and an organisation’s strategic goals, could provide a risk of process inefficiency and missed growth opportunities. The goal of this research is to obtain a better understanding of how LM could contribute to strategic objectives. In our research, we adopted the Congruence Model (ConModel) as a conceptual lens to guide the analysis and structure our findings.^16^ The ConModel, rooted in system thinking and systems theory, outlines the essential elements of organisational functioning. The model can be used as a framework for understanding inner organisational design for strategy implementation and has been successfully applied to the healthcare setting.^17^

In our research, we are especially interested in the main elements of the model: tasks, people, formal organisation and informal organisation. People refer to individuals’ skills, knowledge and motivations, while tasks involve the work and responsibilities required to achieve goals. The formal organisation encompasses official structures, systems and processes, such as roles and policies, whereas the informal organisation includes unwritten norms, values and relationships that shape behaviour. The ConModel emphasises that organisational performance improves when these elements are in alignment. Misalignment, such as tasks not matching people’s abilities or formal systems clashing with informal culture, can hinder effectiveness. Achieving congruence ensures smoother operations and better results.^16^

The problem at the heart of this research relates to broader trends in healthcare, where cost-efficiency and strategic alignment are critical elements. Hence, understanding if and how LM aligns with broader organisational strategies and contributes to long-term healthcare goals is an imperative research pursuit, with the potential to transform healthcare delivery, guide resource allocation and facilitate the transition towards sustainable healthcare practices.

The research question for this study is: *How do Lean Management practices align and contribute to strategic objectives in a healthcare context?* The aim of this research is to explore how LM practices align or can contribute to strategic objectives in a healthcare context.

## Methods

A qualitative research design guided by the Standards for Reporting Qualitative Research was adopted to provide an explanation and practical insights regarding LM practices and strategic objectives.^18^ We performed a qualitative case study with embedded units following an interpretivist paradigm that builds on an extensive qualitative data set. This type of study is suitable when the research seeks to explain a present circumstance, in this study the alignment between LM and strategic objectives.^19^ Our study was guided by a conceptual lens, the Congruence Model (ConModel).^16 17^

 In our research project, we used generative AI (GenAI) for specific, limited tasks. OpenAI’s ChatGPT-4 was employed to refine the manuscript’s spelling and grammar, as well as to develop an initial template for our interview questions. We consulted GenAI to clarify spelling or usage of English terms, revise short paragraphs that required improvement in structure or clarity and generate a foundational structure for our interview guide, which we then adapted for our needs. GenAI was not used to analyse any data, and it did not provide input or generate any of the results in this study.

 In order to highlight any biases or preconceptions that might exist in the research team, we provide the positionality of the team members in this paragraph. The main researchers in this study (OR and KA) have significant experience with research aimed at LM in healthcare. In addition, OR holds a managerial position in education and KA holds a position of director in a healthcare setting. As a result, they are familiar with issues that arise when managing a complex environment, which informs their reflections of observations in the field. Researchers AZ and FV were completing their masters’ degree during this study, and both have a background in business studies. Both AZ and FV had limited exposure prior to this research to the managerial side of the healthcare context. Senior researcher LP has a background in nursing education, practice and research, and has rich experience in qualitative research methods in healthcare.

### Study design

This research applied a qualitative research design with semi-structured individual interviews with healthcare professionals in a university medical centre in the Netherlands. It employs a combined inductive and deductive approach. The ConModel structured the interviews and guided the analysis. The inductive approach immerses researchers in the hospital setting.^20^ The overall design of the study has been sanctioned by the Ethical Board of the Faculty of Economics and Business at the University of Groningen (IRB) (reference: #FEB-20231020–14427).

### Patient and public involvement

Patients and the broader public were not involved in the development of this research project.

### Setting

As we are interested in LM practices alignment and contribution to strategic objectives in a healthcare context, we require a setting where LM is actively applied and linked with organisational strategy. Hence, the rationale to study a large university medical centre in the Netherlands mainly relates to the fact that it has been actively applying LM practices for well over 10 years. The medical centre formulates strategic goals every 5 years and has an in-house LM training programme. To study to what extent process improvements from the LM programme contributed to strategic goals, this research focuses on a subset of long-term organisational objectives. Specifically, we follow projects in the domains of being healthy at work (labelled Sustainable Vitality, which means that employees enjoy going to work and that there is a good work-private balance) and quality improvement (labelled Improving Quality Care).

### Data collection

The study focused on six departments: Kidney and vascular surgery, Care core movement, Clinical department, Systemic diseases, Oncology, Care core Hepato-Pancreato-Biliary disease (HPB), which were all part of one business unit. Primary data consisted of 22 interviews conducted over a period of 4 weeks using a semi-structured interview protocol, site visits and personal research notes (made during and after meetings). Participants were purposefully selected based on their organisational roles and LM experience and were approached via email. Respondents had to be involved in LM practices at the hospital, which meant they had to understand LM and have passed at least one LM training programme, or they should be enrolled in a training course during the research.

All interviews were conducted with two interviewers present (AZ and FV) both on-site as well as digitally. Interview sessions took place in private, undisturbed settings (eg, closed offices or reserved meeting rooms) to ensure confidentiality. All interviews were audio-recorded on a research-use smartphone and/or laptop with participants’ consent and transcribed verbatim (ie, including false starts) to preserve the authenticity of responses. Transcripts were not edited for grammar or clarity to ensure accuracy in qualitative analysis. Interviews were conducted in Dutch, and only citations used in the manuscript and codebook were translated to English. All audio recordings have been deleted after completing the transcripts and transcripts remain securely stored, accessible only to the research team, and have not been shared with the case site or external parties.

The two interviewers setup enhanced the validity of the obtained data, and the interviewers did not establish a relationship with the respondents before the interviews were conducted. This setup minimised the risk of interpretation differences, enabling mutual verification of observations and comparison of notes. In our study, saturation was reached after completion of approximately 18 out of the total of 22 interviews. After the 18th interview, no new insights emerged, indicating sufficient coverage of the study topics. At the start of the interview, the respondents were instructed on the objective and goals of the research project. All interviewees signed an informed consent before participating. An overview of the respondents has been added to table 1.

**Table 1:** Respondents of the interviews

Job title	Department	ID#
Coordinating nurse	Kidney and vascular surgery	01
Team manager		12
Coordinating nurse	Care core movement	02
Coordinating nurse		03
Nurse		04
Coordinating nurse		05
Coordinating nurse		06
Coordinating nurse		08
Nurse		20
Team manager	Clinical department	10
Team manager	Systemic diseases	14
Team manager		15
Nurse & quality nurse	Oncology	17
Team manager	Care core HPB	18
Team manager		19
Management consultant	Business Unit	13
Management consultant		11
Management consultant		07
Director		16
Quality consultant		09
Educational advisor		21
Management consultant		22

Secondary data was used to obtain a better understanding of the LM approach, long-term objectives of the organisation and the course of action that was envisioned. Secondary data consisted of a variety of materials, as shown in table 2. All the primary and secondary data were used in the analysis during this study. During the course of this research, researchers AZ and FV conducted around 10 site visits to gain a better understanding of the organisation and its context.

**Table 2:** Overview of collected data

Type	Source	Size	Purpose
Primary data	22 Interviews	~990 min of audio data	To gather employee insights and experiences with LM working
	Transcript interviews	258 pages	To analyse the data
	Exploratory meetings	Three both on-site and digital	To obtain an understanding of LM practices in the organisation
	Personal notes	~50 written pages	To obtain an overview and better understanding
Secondary data	Policy document	22 pages	To build awareness of the strategic objectives of the organisation
	Annual report of 2022	215 pages	To obtain a better understanding of the mission, vision, strategy, etc.
	Documents on one of the LM Improvement projects	PowerPoint slides	To build an understanding of what working with A3 reports looks like
	Documents on the improvement approach of the organisation	PowerPoint slides	To get an understanding of how the organisation approaches improvement
	PowerPoint about how the organisation thinks about continuous improvements	PowerPoint slides	To gain insights into how improvement projects are addressed in the organisation

Interview protocols were developed with the aid of GenAI. Here GenAI generated an initial template, which was then refined by the research team (AZ, FV and OR). The interviews covered the elements of the ConModel, including tasks, informal organisation, formal organisation and individuals, and included 9 general questions, 23 main questions and 4 closing questions. Separate protocols for the director and operational staff were chosen to capture their distinct perspectives and roles within the organisation, providing a comprehensive understanding of how LM practices contribute to strategic objectives in healthcare. The interview protocols have been added to the online supplemental appendix A.

### Data analysis

Transcripts were read again to eliminate any remaining errors, and Atlas.Ti was used for data management and analysis. This study adopted a cyclical process, moving between three coding methods: open coding, axial coding and selective coding.^21 22^ The coding process was performed by AZ, and in the first phase, 52 open codes were created and linked to the transcribed interviews using Atlas.Ti. During axial coding, key themes and categories were analysed, and the 52 open codes were grouped into seven axial codes. During the coding process, there were regular meetings where AZ, OR and FV discussed the codes and related findings. After identifying the main themes, similarities and differences were examined and compared. Finally, the analysis highlighted how these themes and categories contribute to answering the research question. The complete codebook is available in the online supplemental appendix B.

## Results

This section presents the findings of our study. First, we present the main insights per element of the ConModel. Then we discuss the implications for strategic alignment and present an updated framework incorporating the main lessons from our research.

### Tasks

LM is seen by most respondents as valuable for process improvement. However, when asked about the influence of LM on tasks and responsibilities, respondents reported that LM influence was limited. A nurse (ID01) reported: “*Yes, we have a little less stress related to beds being available as the VODs* (Provisional Discharge Date) *are more correct. I think that’s the biggest improvement in terms of tasks. You don't have to move* (patients) *around as much*”. It seemed that there was limited follow-up and integration in daily practice after formal LM-training. Respondents indicated that they did not engage with LM, apart from their personal improvement projects, and here time and resource constraints are the main barrier towards LM impacting the overall strategy. When asked about the impact of LM on healthcare quality, most respondents reported positive effects. Examples included faster patient discharge, improved medication safety and increased bedside time for nurses. As one respondent noted: *“We really see an improvement (…) the nurse is more present in the room, so they have more contact with the patient”* (ID07). Overall, the main challenges as reported by the respondents relate to time constraints, limited resources and the need for ongoing support and guidance. To maximise the benefits of LM, there is a need for a more comprehensive approach that addresses these challenges and integrates LM principles into daily operations.

### Informal organisation

Respondents reported mixed experiences when reflecting on support and encouragement from management to establish a culture of continuous improvement. Nurses were critical, with statements such as: *“They* (management) *don't encourage* (a culture of improvement)*”* (ID06). However, respondents with management responsibilities were more positive, although they had similar experiences: “*As a manager, I do encourage it* (a culture of improvement) *a lot. Only my manager does not encourage it*” (ID10). Respondents mention that there are different cultures across departments and that an open and collaborative culture should facilitate LM projects.

When asked about the opportunities for suggesting process improvements, respondents indicated that the opportunities were there. Some respondents mentioned that time for contributing to improvement projects would be a reward in itself, and indicated that appreciation as a reward would also be valuable. A staff member said: “*Appreciation is super important*” (ID15). Hence, while respondents apparently do not experience a culture of improvement, they acknowledge that structures to address improvement exist, although there is a perceived lack of management support for continuous improvement, which might hinder the effectiveness of LM.

When asked about the alignment of LM with the organisational culture, multiple respondents saw a good fit, emphasising the culture’s openness to innovation and result-oriented focus. Respondents highlighted the importance of building employee support and providing training, such as Green Belt programmes, to facilitate LM adoption.

### Formal organisation

Generally, respondents had limited knowledge of the strategic goals of the organisation, with nursing staff being unable to provide examples. Related to LM being able to contribute to strategic goals, opinions varied. Some respondents felt that LM could contribute while others did not see value at all. According to respondents, information on the alignment between strategic goals and LM projects was lacking. While they indicated that there are many channels to share information, such as mail or intranet, not everyone regularly visits each channel. One of the staff members said: "*There are a lot of communication channels (…) but not everyone reads them every day. There is so much information, but you actually just can't keep up* (…)” (ID15). Overall, respondents lack awareness of strategic goals, with varying levels of engagement, particularly among nurses. Opinions on LM’s impact on engagement differ. Communication challenges exist, and there’s a desire for better information flow, suggesting increased visibility of strategic employees on the work floor. Positive feedback on LM support but limited organisational backing is noted. Despite LM projects, autonomy perception remains unchanged.

Regarding the fit between LM and the organisational structure, responses varied. Many at the operational level admitted to lacking awareness of the organisational structure. For instance: *“As a nurse, I have no idea about the corporate structure within the organisation”* (ID01). In contrast, those in higher-level roles, such as Management Consultants and Team Managers, viewed LM as highly compatible due to its clarity, scalability and result-oriented nature. One consultant explained: *“It’s a clear methodology (…) you can apply it on a small or large scale, making it very effective”* (ID22).

### People

When asked about the skills, knowledge and attributes necessary for LM work, respondents highlighted several key areas. In terms of knowledge, familiarity with Green Belt certification and Lean methodology was commonly mentioned. Two nurses emphasised the importance of college-level education to grasp LM’s distinct way of thinking. As one respondent stated: *“I think you really do need a college level of thinking”* (ID04). Regarding skills, respondents frequently mentioned perseverance, discipline, and the ability to address others as critical. Additional skills included creativity, openness to innovation, collaboration, patience, listening and critical thinking. For example: *“You have to have discipline and perseverance”* (ID19). However, some respondents expressed uncertainty about the prerequisites for LM work, suggesting that a passion for the topic could suffice. As one nurse explained: *“I don’t think it matters that much (…) you just need to be really behind your topic”* (ID02). Regarding personal development, respondents frequently mentioned that LM fostered a new way of thinking. One nurse reflected: *“You get a whole different mindset, and I like that a lot”* (ID03). Opinions on LM’s contribution to strategic goals were mixed. Some respondents saw clear alignment between their projects and organisational priorities, such as medication safety and workload reduction. For example: *“With my Lean A3, we are trying to reduce the workload (…) so there is less pressure on the beds”* (ID06). Others, particularly Team Managers, expressed scepticism. One respondent stated: *“The strategic goals are still too high-level to run a Lean project on”* (ID07).

### Bringing it together: LM and strategic objectives

Opinions on LM contribution to strategic goals are divided. Some respondents identified direct contributions, such as improved medication safety and workload reduction, while others remained sceptical, perceiving limited connections. Despite this, LM is widely seen as a good fit with the organisation’s innovative and results-oriented culture. Respondents emphasised the importance of employee engagement and training, such as Green Belt programmes, for the successful implementation of LM. However, a lack of familiarity with the organisational structure, particularly at the operational level, was noted as a challenge. While LM’s link to strategic objectives remains unclear for some, its impact on healthcare quality is widely recognised. Respondents reported positive outcomes, including faster patient discharge, improved medication safety and increased bedside time for nurses. Additionally, LM was seen as contributing to personal development, fostering a new mindset and improving problem-solving abilities. Overall, LM demonstrates clear benefits for healthcare quality and staff development. However, to strengthen its alignment with strategic goals, improved communication, awareness, and active efforts to embed LM into the organisational culture are needed. Despite some scepticism, tangible examples—such as reduced workloads and enhanced safety—highlight LM’s potential to support strategic objectives. Table 3 provides an overview of the main causes we identified in our study that influenced the contribution of LM on strategic objectives.

**Table 3:** Causes that influence LM’s contribution to strategic goals

Positive	Negative
Positive experience with LM; Positive impact on healthcare quality; Improvement projects contributed to strategic objectives; A reward system could contribute to the engagement of employees to foster a culture of continuous improvements.	Lack of resources, time; Lack of communication strategic objectives; Minimal support from management for culture of continuous improvement; Receiving LM training from different sources; Experienced lack of appreciation from management.

LM, lean management.

Following the results, we can identify a number of facilitators and barriers for LM. In table 4, one can find the barriers and facilitators for implementing LM mapped across the different elements of the ConModel.

**Table 4:** Facilitators and barriers, linked with the Congruence model

Elements congruence model	Facilitators	Barriers
Informal organisation:	Fostering a culture of continuous improvement (this research found that a reward system could stimulate a culture of continuous improvement).	Lack of employee engagement (a nurse mentioned that the course felt like something that was obligated).
Tasks:	Provide adequate training (Green belt or Black Belt).	Limited knowledge of LM practices.
		Not making resources available (there was no time available to work on the A3 reports).
Individuals:	Committed managers (most staff members indicated that they receive support from their management).	Lack of management support (most of the nurses missed support from their managers).
Formal organisation:	No hierarchical structure	The nurses mentioned a lack of communication of strategic objectives.

LM, lean management.

## Discussion

This research aimed to provide an answer to the following research question: “*How do Lean Management practices align and contribute to strategic objectives in a healthcare context?*”. To the best of our knowledge, this is one of the first studies focusing on LM and its alignment with strategic objectives in a healthcare setting. Results show that LM can align with strategic objectives through concrete improvement projects. However, our results also indicate that the contribution of LM to the overall strategy, in our current study, is limited. We find that common barriers, such as a lack of management support and employee engagement, affect the potential contribution of LM to the overall strategy.

In our study, we applied the ConModel as a conceptual lens, and this perspective allowed us to map the various facilitators and barriers across the models’ functional elements. The findings indicate that the employees could not provide an example of the strategic objectives and that most interviewees on the operational level could not provide a description of the culture or structure of the organisation. This indicates a misfit between the people and the structure, and between the people and the culture. In addition, this might raise questions as to what extent we can expect LM initiatives to align with broader organisation-wide strategic objectives.

In this study, we found that nurses, as front line employees, often have different experiences of LM compared with staff with leader or management responsibilities. While nurses recognised that LM could improve their tasks and allow them to spend more time with patients, their attitudes and willingness to adopt organisational changes were influenced by limited staff engagement, support, education and communication. To address these challenges, it is important to actively involve and engage frontline staff in LM design process across disciplines and hierarchical levels.^15^ A more holistic, multistakeholder view of value in healthcare is needed to ensure LM integration with daily practice and create improvements that benefit patients, clinicians and management. Early involvement of all stakeholders through different methods (eg, teamwork, meetings, workshops, open communication) can help optimise the outcomes.^23^

This research found that LM practices contribute and align minimally to strategic objectives due to the found misfits and also due to the many barriers and challenges that implementing LM entails, which is also supported by other research. The research of Kunnen *et al*^15^ found similar barriers in their study, such as a lack of follow-up from projects, not making resources available to personnel and leaders who do not acknowledge the value of employees. Furthermore, the study of D’Andreamatteo *et al*^8^ indicates barriers, such as a lack of leadership commitment and support, and employee engagement, which were also found in this research. Moreover, our results fit with the findings of Burgess and Radnor who show a limited number of hospitals taking a systemic approach to LM, which underlines the challenges related to aligning LM and strategic objectives.^24^

The results underscore the critical role of effective facilitators in successfully implementing LM practices. The findings suggest that LM can contribute meaningfully to the strategic objectives of healthcare organisations. However, achieving this contribution requires several enabling factors. Organisations must equip employees with the necessary skills and knowledge to work effectively with LM. Employees also need sufficient time to engage with and execute LM projects, along with consistent support from management throughout the process. Additionally, organisations should allocate adequate resources to LM initiatives, and the management must recognise and appreciate employees’ efforts. By addressing these factors, organisations can reduce barriers to LM implementation and resolve misalignments between organisational elements, thereby maximising the impact of LM on strategic objectives.

### Theoretical implications

LM in healthcare has become a growing topic of interest both in the healthcare world and the academic literature over the last decade.^25^,^27^ The research adds nuance to the understanding of LM’s impact on strategic objectives.^28^ The study identifies barriers such as a lack of resources and support as key impediments to realising the full potential of LM in healthcare organisations. These barriers were also found in the study of Kunnen *et al*^15^ where barriers and facilitators were shown to be important in the sustainment of LM practices. In addition, our findings align with the work by Hung *et al*,^29^ who emphasise the importance of leadership support and organisational culture. Moreover, the managers who want to implement LM could use the Congruence model to get a better understanding of the organisation and the alignment between the factors.

Theoretical implications also emerge regarding the operational definition of LM healthcare management proposed by Rotter *et al*.^30^ The study underscores the importance of organisational support, employee skills and management appreciation as critical facilitators for LM practices to align and contribute meaningfully to strategic objectives. These factors, when addressed, can reduce barriers and enhance the overall effectiveness of LM implementation.

Based on the main findings, this study has two propositions aimed at those that have control in healthcare environments:

1. Leaders in healthcare organisations should be aware of the barriers and challenges that LM implementation entails and they should facilitate the organisation and their employees with enough resources and time to foster a culture of continuous improvement.
2. Leaders in healthcare striving for a culture of continuous improvement during LM implementation should prioritise managerial support, resource allocation, appreciation and transparent communication, identifying and rectifying any incongruences among these elements.

### Limitations and future research

This research was conducted within a specific context—an academic university hospital. Although this organisation is one of the largest academic hospitals in the Netherlands, the findings may not be generalisable to healthcare organisations in other contexts. To enhance the applicability of these results, future research could explore other hospital departments and healthcare services in diverse settings, enabling broader validation of the findings. In addition, qualitative research is always susceptible to researcher bias, our perceptions and experiences might have influenced our analysis. In our study, we tried to mitigate the effect by having multiple researchers involved in the analysis and through the addition of a conceptual lens. Additionally, the study identified distinctions between nurses and other employees. However, it did not specifically investigate differences between nurses and employees operating at a strategic level. Future research could delve into the roles and contributions of various employee groups to strategic objectives to provide deeper insights into their unique impacts. Moreover, future studies might take our applied methods and use the ConModel to assess connections between strategy and frontline actions.

### Implications for practice

The identified findings can be employed by healthcare practitioners who are looking to implement LM in their organisations. Healthcare professionals should be aware of the barriers and challenges that LM implementation entails and they should facilitate the organisation and their employees with enough resources, time and training considering individuals’ needs. This research highlights the importance of appreciation from the management to foster a culture of continuous improvement. In order to achieve this, organisations should focus on managerial support, providing enough resources, appreciation and open communication. This research also reaffirms key success factors identified earlier,^8 13^ emphasising the significance of committed managers, strong leadership and organisational readiness for effective LM implementation.

### Conclusion

This research explored how LM improvement initiatives align with a hospital’s strategic objectives. The findings revealed several challenges, such as a lack of resources or a gap in translating strategic goals to work floor activities. The contribution of LM to the organisational strategy is likely to be limited when barriers, eg, lack of follow-up on the project, are present. Ultimately, our study highlights several facilitators and barriers linked with the various elements of the congruence model. Future research might use our findings to further our understanding of how a complete LM approach, embedded in all organisational elements, links with overall organisational strategy.

## Supplementary material

10.1136/bmjopen-2025-099758 online supplemental file 1

## Data Availability

The data that support the findings of this study are available from the corresponding author upon reasonable request.
