# Supplementary material for: Alignment and contribution of lean management practices to strategic objectives in a healthcare context: a qualitative study in a university medical centre
Source: BMJ Open. 2025 Dec 15;15(12):e099758. doi: 10.1136/bmjopen-2025-099758 (PMC12706196; doi:10.1136/bmjopen-2025-099758)
Supplement: online supplemental file 1 [file bmjopen-15-12-s001.docx]

**APPENDIX A: INTERVIEW PROTOCOL**.

*Note: original interview protocol was in Dutch.*

**PROTOCOL 1**

**General questions**.

We start with a series of general questions; these are mainly about your work and experience within the organization.

1. What is your year of birth?
2. What position do you hold within the organization?
3. At which department do you work?
4. How long have you been working at the organization?
5. What are some of your main tasks and responsibilities?
6. Which Lean improvement project have you worked on or are you still working on?
7. Can you briefly state what your involvement is or has been in the Lean improvement project?
8. How many Lean improvement projects have you worked on in total?
9. What kind of training or education have you had on Lean working?

**Main questions**.

We have now arrived at the central questions of our research; these are mainly about the organizations' strategy and the Lean project. We speak here of strategic goals; strategic goals are goals that the organization wants to achieve in the long term. Often, we speak of time frames of several years. We have divided the main questions into a number of topics, namely 'tasks', 'informal organization' and 'formal organization'.

Tasks.

1. Can you briefly describe what Lean working is within the organization?
2. How has the new way of working, i.e., the way of working after the Lean improvement project, affected your tasks and responsibilities?
3. In what way has the organization facilitated in developing the skills and knowledge needed for working with Lean?
4. To what extent do you adhere to the new way of working?
5. Can you give examples of concrete improvements in the tasks you perform daily as a result of the Lean project?
6. What challenges do you experience in applying Lean in your daily work?
7. How have you experienced Lean yourself?

Informal organization.

1. How would you describe the organizational culture?
2. How would you describe the department culture?
3. To what extent does lean working fit into this?
4. To what extent is a culture of continuous improvement within the organization encouraged and supported by management?
5. In what way can organizational culture add value in the implementation of Lean improvement projects?
6. What are currently the possibilities for bringing in suggestions for process improvement in your department?
7. What reward systems are used within the organization to encourage employees to make contributions to continuous improvement?
8. None: would it add value?

Formal organization.

1. In what ways do you think Lean working fits into the organizational structure of the organization?
2. How do you see the impact of the way of working Lean on the quality of care provided by the organization?
3. Can you give some examples of strategic goals appropriate within the organization?

If someone cannot give an example, explain the strategic goal (Vitality) and make it concrete. Then the respondent will know what it is about. You can tell yourself that the goal was not known, but maybe they will know it once it is described more specifically.

In addition to appreciation, we think it is important to focus on vital employees. Employees who feel vital work with pleasure, are better able to withstand work pressure and are optimally employable.

1. How do you feel involved in achieving the organization's strategic objectives in your daily work?
2. And how does Lean contribute to this involvement?
3. How is information about the strategic goals and Lean projects communicated to employees at the operational level?
4. How can you feel better informed?
5. In what way do you think the Lean project contributed to achieving the strategic goals?
6. To what extent have you had support with the Lean projects and the new way of working?
7. In what way does Lean working contribute to your personal development?
8. Can you explain how the hierarchical structure has been in the approach to the Lean project?
9. In what aspects of your role or work has autonomy changed after the implementation of Lean?
10. In order to work lean, what skills & knowledge & attributes would you need for this?

**Closing questions**.

Here we come to the last questions of our interview in which we discussed Lean working and strategic goals.

1. Are there things we did not discuss that you would still like to mention?
2. Do you have any suggestions for questions we can add to improve our interview?
3. If we have additional questions at a later time, may we contact you briefly? Perhaps for clarification we may send you a short email? <mail query>
4. Is there a colleague you think we should definitely speak to as well?

Thank you very much for your cooperation in our interview. If you have any questions or comments at a later time, please feel free to contact us at <mail/phone>. If you want, we can send you our final thesis, are you interested in that? <If Yes, include mail and make sure the final result goes to the respondent>.

**PROTOCOL 2**

**General questions**.

We start with a series of general questions; these are mainly about your work and experience within the organization.

1. What is your year of birth?
2. What position do you hold within the organization?
3. How long have you been working at the organization?
4. What are some of your main tasks and responsibilities?
5. How many Lean improvement projects have you worked on in total?
6. What kind of training or education have you had on Lean working?

**Main questions**.

We have now arrived at the central questions from our survey, these are mainly about the organizations’ strategy and Lean.

1. Can you briefly describe what Lean working is within the organization?
2. How would you describe the evolution of Lean within the organization, from its introduction until now?
3. In what way do you think Lean contributes to the achievement of the strategic goals?
4. How would you describe the organizational culture?
5. To what extent do you think employees are aware of the strategic goals?
6. What kind of communication methods are in place to create awareness among employees regarding the strategic considerations?
7. How does the organization measure the successful integration of the outcomes of the Lean projects?
8. What factors have contributed to effective implementation of Lean projects?
9. Is there a check for alignment of strategic goals and Lean projects, so e.g. is alignment actively managed or is there some other mechanism?
10. How do you see the role of Lean projects evolving in the future strategic direction of the organization?
11. Are there specific areas where you believe Lean methodologies can be used even more effectively to achieve strategic goals?
12. How is employee feedback collected and used to improve Lean projects and strategic considerations?

**Closing questions**.

Here we come to the last questions of our interview in which we discussed Lean working and strategic goals.

1. Are there things we did not discuss that you would still like to mention?
2. Do you have any suggestions for questions we can add to improve our interview?
3. If we have additional questions at a later time, may we contact you briefly? Perhaps for clarification we may send you a short email? <mail query>
4. Is there a colleague you think we should definitely speak to as well?

Thank you very much for your cooperation in our interview. If you have any questions or comments at a later time, please feel free to contact us at <mail/phone>. If you want, we can send you our final thesis, are you interested in that? <If Yes, include mail and make sure the final result goes to the respondent>.

**APPENDIX B: CODEBOOK**.

| **Axial code** | **Open code** | **Explanation when the open code / axial code is used** | **Quote from the data illustrating the open code / axial code** |
| --- | --- | --- | --- |
| **Tasks** | **Definition LM** | When the data indicates a definition of LM | **Respondent 14:** “I see it more as a tool to better analyze your problem and actually come up with where exactly is it? Because we think way too big, so you actually start filtering it out and making it smaller and then eventually you have, yes the base or the bottom and if you have the bottom, then you can move on”. |
|  | **Influence LM on tasks & responsibilities** | When the data indicates the influence from LM on the tasks and responsibilities | **Respondent 15:** “Yes I think more awareness in the sense of aha, a subject is being scrutinized and then it is kind of nice that there is an awareness in that and that they see that, but that we see that as well”.  **Respondent 22:** “Indeed, I think for me it's also really a different way of looking at it. It's true, I think with you there's a lot of focusing from that thought that indeed you go out on the shop floor. That you're going to look, that you're not going to do things by feel, but that you say well, this way and that you're going to draw it out, that you're going to measure it”. |
|  | **Facilitation** | When the data indicates the way in which the organization facilitates the employees with the skills needed for working with LM | **Respondent 4:** “I think that's mainly by offering the course. Uh, that you have the opportunity to do it. And that they do have the right people in there to train you in that? Uh who do want to help you improve. But you also have to say that indeed the moment you have completed it, that it stops there. Then it stops”.  **Respondent 5:** “Yes that we were allowed to take the Green Belt. We then even have the Yellow Belt now. That's for the caregiver, the Orange Belt for the work groups so to speak. And then Green Belt is actually for directing nurse or uh management. So now a yellow belt has been started for those caregivers who walk along. So that's all being arranged from ***name organization*** as well”.  **Respondent 18:** “Yes, we got of course, all we got was that yes, that training that Green Belt and now well then, that Orange Belt. But we do have to get ourselves there and it then gets organized for us, but there are no handles otherwise. We have to arrange everything that it but to make time for it or to give your team time for it. Yes, you have to arrange that yourself”. |
|  | **Examples of improvements** | When the data indicates examples of improvements that were caused by the LM projects | **Respondent 6:** "Uhm well, as a result of the projects that have been run, so to speak. So you have a piece of medication safety, a lot of steps have been taken. Uhm by now there are also new medication carts, which was another part of that project, just like posters about the collection process and the distribution process of medication. Uhm also the IO transfer and that is being started up now and those are all things that came out of a lean project".  **Respondent 8:** "Well, in any case, for students and new employees, well, that coincides a bit, those induction plans as well. Uh, you always have to make a lot of arrangements for the first day, but now that it's all sorted out and people know where they have to be, we don't have to do everything anymore, it's easier". |
|  | **Challenges implementing LM** | When the data indicates experienced challenges for implementing LM | **Respondent 15:** “Yes, what I just said before is that sometimes you don't realize that it's because of that, but it's because of something completely different. That, of course, is a very nice awareness of the problem”. |
|  | **Compliance working with LM** | When the data indicates whether or whether not the respondents still work with LM | **Respondent 19:** “Uhm, well, I do think in my leadership role I do give some guidance on that. So you always keep it in the back of your mind. But sometimes I find it difficult, you quickly revert to old patterns, but I also notice that, yes, everyone really does their best. So yes, a bit like that”. |
|  | **Personal experience LM** | When the data indicates how the employees experienced LM | **Respondent 15:** “I think it is very effective, but I do think it is a very, no difficult is not the right word. A time-consuming process where people actually drop out very quickly. And that's because the nurse yes who works on the floor, you know. They do want efficiency, so that processes become more efficient, they do favor that. But I feel like it's a time-consuming thing. And especially if you don't get time to work it out, they're just not going to keep that motivation. I think that's a real shame and I've experienced that myself”. |
| **Informal organization** | **Description organizational culture** | When the data indicates a description of the organizational culture | **Respondent 5:** "Big. And if you then look at Lean, I know what my colleagues are doing, but I don't know what the neighbors are doing, for example. So, I still miss that".  **Respondent 18:** "I find that a very tricky one, because each department really has its own culture. Yes, that really is such a world of difference". |
|  | **Department culture** | When the data indicates a description of the department culture | **Respondent 14:** "There is a really united and open culture in our department".  **Respondent 15:** “Warm, open, cohesive”. |
|  | **LM & organizational culture fit** | When the data indicates a fit between organizational culture and LM | **Respondent 17:** “Yes, I think in itself it is good because you also, yes you have to look at your problem from different angles. So, in that respect I do think in such a large organization it is very relevant to use it, but it is. Yes, if you really do it on a practical level. Yes, then I actually also think it's a good way because you learn to think very differently than just doing it instead of really getting into the rationale or the problem. So, I think lean fits within the culture”.  **Respondent 18:** "Yes, with this culture it fits well, I think. Yes fine. It gives you a handle to also focus on problems that are there, that can be improved". |
|  | **Support management culture continuous improvement** | When the data indicates support from the management for a culture of continuous improvement | **Respondent 18:** “Look, by us, by us as managers, we as managers encourage that and really try to give space for it. But I don't think that from *name organization*. There everything is mainly thrown down very much and so also a bit over the fence and we have to deliver to the *name organization*. And we only have to do this and we only have to do that, while we also have to care for patients and that is sometimes forgotten. It's a bit tricky because if you ask the Executive Board, they also say that PDA cycle. So, they are very much of the opinion that that should be it, so to speak. They don't feel that they are throwing it over the fence, but because there are some layers in between, that is also the case”.  **Respondent 1:** "Not so much, I think that has to do with several factors. We have staff shortages everywhere on every department anyway so you'd rather have nurses on the floor than working on improvements. Yes, in your time for Lean is just not there, so that is not supported that much by several factors." |
|  | **Reward systems** | When the data indicates the reward systems used in the organization | **Respondent 1:** "I think if you get time for it that's a reward in itself".  **Respondent 6:** "Mm hu. To my knowledge not really anything. No actually".  **Respondent 8:** "No? No idea. No". |
|  | **Added value reward systems** | When the data indicates added value of reward systems | **Respondent 1:** "Yes maybe yes. Yes, yes, yes, a bit of reward. It is a bit of appreciation for what you have done. Uh, we now have one of *** we did get a lot of appreciation because he really did uh encourage and support a lot and that was really nice. And uhm and you also saw that when he was here saying interim. Such a positive approach. And then we got lunch. It only has to be something small, sometimes an ice cream. And uh sometimes you also see, yes this is a bit different. But for example, with sick people. Uh well they might have been on sick leave for two years. Yes, uh you run yourself out of the snot for that person".  **Respondent 12:** "Yes, but appreciation, and I even notice this in my own group, in the group of managers, expressing appreciation uh really doesn't happen much, and we do try to do it in the department. And yet you often get bogged down in giving feedback, but not mentioning the extra positive and that remains difficult". |
|  | **Added value culture in LM** | When the data indicates added value from the culture for the LM projects | **Respondent 10:** "If you have an organizational culture of keep looking critically at yourself, keep looking critically at the processes. Focus on going through that whole PDCA, I think if that would get more attention from central, then the awareness might also be easier, also at individual level or departmental level to bring back."  **Respondent 12:** "Well, by rolling it out a bit better, I think. It's often just of here you have the project and have fun with it. And uh look, now in Lean of course you are included, but there are plenty of projects uh where you only get half the information or that you hear afterwards that a project is running, that you don't even know about it, that one department is working on it, but the other department isn't, and then everyone has their own interpretation. I think clarity is something that is important. |
|  | **Suggestions process improvement** | When the data indicates the possibility to bring in suggestions for improvements | **Respondent 12:** "Yes, it is open. As far as that is concerned, we are just open to that then, especially with the new regies of course now we are anyway looking at everything and uh taking stock again, so that is open".  **Respondent 2:** "For me this is very easy because now I am actually the director here. So, I get an office day once in a fortnight every week. So, I have the time to pick things up, to check things, to deal with problems that I see myself or that someone suggests to me. Uh but I think when you're on the shop floor yourself you really have to be very decisive and enthusiastic and motivated to take it up yourself. Oh, you have to put yourself down with us. Yes, uhm to do something with that? Yes, and your management will always say hey, hearty. Good, so nice, nice doing it. But you just don't get time for it yet".  **Respondent 6:** "Uhm yes. Good question. Maybe there is no concrete there actually. I think we do try to spread the word that, hey, if there are certain domain groups uh from the direction nurses that if there are things, they can reach us. But I don't feel that we're very much alive yet either because it's just really very new. Uhm. That maybe that could be a way. Yes, but that's not really a way yet at the moment". |
| **Formal organization** | **Autonomy** | When the data indicates a change in the autonomy of the employees after the LM projects | **Respondent 6:** "Not really. I think actually. No, no, no. No, I. I don't feel that's more or less".  **Respondent 8:** "Hm. Not very much, no".  **Respondent 11:** "No, not in my case". |
|  | **Involvement in strategic objectives** | When the data indicates to what extent respondents feel involved in achieving strategic objectives | **Respondent 4:** "Really difficult, yes. Because I do enjoy coming to work, but I don't necessarily feel that ***name organization*** is doing a lot about it. Uhm, I think I just have a nice job because I have a nice team and really like the patient category. So, I don't know to what extent the board contributes to that".  **Respondent 6:** "Well not really one at all actually. I don't necessarily feel involved in when I think about what they do for a good work-life balance. Yeah, I don't really notice that much of that effectively. No actually". |
|  | **Contribution LM involvement** | When the data indicates whether or whether not LM could have an influence of the involvement in achieving strategic objectives | **Respondent 8:** "Making more visible than maybe, making that process visible".  **Respondent 15:** "Do think so, if you were to make it small first, why don't we feel involved? Is because then once you are asked and then you are not and then there is no structure in it at all". |
|  | **Contribution LM to strategic objectives** | When the data indicates how employees feel about their contribution for achieving the strategic objectives | **Respondent 6**: “Well not at all really. I don't necessarily feel involved when I think about what they do for a good work-life balance. Yeah, I don't really notice that much of that effectively. No actually.’’  **Respondent 18**: “And you do have vitality weeks, but it's mainly focused on the people who work here in the office in the A tower. But it's not for nurses. They can't sign up for yoga classes at all because they just have to take care of the patients. Their work can't wait. So, they always hit the mark there. And I think that leads to more dissatisfaction towards nursing. You always see, office staff are there, but the nurses are not.’’  **Respondent 19**: “We are not involved by the organization. So, we have a power nap bench for the night shift and things like that. But anyway, in that, the hospital does not facilitate space to put it down. So yes, it should not cost a patient room, so then of course it has to be in an office space, but in theory you can't put a couch or chair there because that's where people work.’’ |
|  | **Contribution LM to personal development** | When the data indicates the contribution of LM to the employee’s personal development | **Respondent 15:** "By just making things smaller. Making notes with okay, I have to buy there and there, for Christmas I have to buy this, buy that. That you make notes so you make things smaller for yourself, so you get peace in your head, so a bit more space and maybe put things into perspective. But I'm not consciously working on it privately, not that I think "oh I'm going to put a Lean on that".  **Respondent 17:** "Well, I think I've said a lot of times, but looking at problems a bit differently. And yes, also because it is such a large organization. Not just. Sometimes you don't just need your department, you need other departments. And the best way to do that too is to just have it clear what your goal is, what a problem is and then be able to ask more focused questions. Yes, if you need them. Yes. Is that still then. |
|  | **Communication strategic objectives** | When the data indicates how information about the strategic objectives and LM projects is communicated to employees | **Respondent 2:** "So I think it's very mediocre too, yes. Uh, because all our posters are hanging here very nicely and that's where it stops. So, we all have very nice ideas and then you either have to send emails or you have to constantly tell your colleagues to think about that or think about that". |
|  | **Hierarchic structure LM projects** | When the data indicates how the structure was experienced was the A3 projects and the cursus. | **Respondent 2:** "Well, we didn't really have very much than the hierarchical structure I thought. That also saves because we don't necessarily have to work with doctors, because we do still have that hierarchy, but stays among colleagues and again to think about because we had the interim manager. Who just sat between us. Who knew what was going on in the department. Who was not, who was not above us either. I am a teacher and you have students. So, I didn't experience that very much". |
|  | **Knowledge strategic objectives** | When the data indicates examples given by the employees about the strategic objectives | **Respondent 2:** "No, I really don't know. But I know quite a lot about my department but about the whole organization actually very little".  **Respondent 3:** "Gee. I can't say so one, two, three".  **Respondent 19:** "No, no idea". |
|  | **LM & organizational structure fit** | When the data indicates how LM fits in the corporate structure | **Respondent 19**: ‘’Yes, I think it does fit in well because it does give nice guidance. But yes, you do need time to work it out. We need to be able to hire more staff so that we also have time and space for improvements.’’  **Respondent 20**: ‘’Well, I don't know that very well because I'm not that much into the organization of course.’’ |
|  | **Impact LM on quality of healthcare** | When the data indicates the impact of LM on the overall quality of healthcare | **Respondent 1:** “I do think the impact is huge though. Certainly, what I just said everyone was doing it for themselves first, so then there were, I'm just saying an example. I don't know if it's true, but there were five departments working on the same problem. So, five people were on it and now one person is working on that problem and they spread that to those five people. Yes so, those other four people, they have time to tackle other things again and brainstorm on other problems. So yeah, yeah. I think the impact on quality is really quite significant”. |
|  | **Support with LM projects** | When the data indicates the received support during the LM projects | **Respondent 17**: ‘’Actually only during the training. Not anymore.’’  **Respondent 6**: ‘’At the time when the whole course was running, quite a lot. Our coach and the management paid quite a lot of attention to it, but when the course was finished, it was finished. And after that you weren't really stimulated or supported much anymore.’’ |
|  | **Skills LM working** | When the data indicates what knowledge, skills and characteristics people need to work LM | **Respondent 5**: ‘’I think it's a difficult question. I don't think there's really an answer to that.’’  **Respondent 7**: ‘’Belt training in any case. But that really has to do with that it is important to make sure that you understand the underlying thought of LM, because it is not just a model, there is really a thought behind it. I think it's just really good to be taken into that and to go through that as well.’’  **Respondent 11**: ‘’I think you could actually do this at any level of education I think.’’  **Respondent 19**: ‘’Decisive. You have to have discipline. Perseverance.’’ |
|  | **Improving information supply** | When the data indicates what could be improvements for better communication ways | **Respondent 17**: ‘’Well, I know there's also a lot of use of the screensavers. That always works pretty well.’’  **Respondent 3**: ‘’Maybe instead of just the screens that people are also more visible. Who's behind it? Or who is setting a good example?’’  **Respondent 5**: ‘’I don’t know that answer either.’’ |
| **Individuals** | **Important tasks & responsibilities** | When the data indicates examples of the employees’ tasks and responsibilities | **Respondent 10:** "Uhm in my current position, that's mainly HR management. Uh yes, responsible for quality-of-care delivery, of care safety". |
|  | **Reward systems** | When the data indicates the reward systems used in the organization | **Respondent 1:** "I think if you get time for it that's a reward in itself".  **Respondent 6:** "Mm hu. To my knowledge not really anything. No actually".  **Respondent 8:** "No? No idea. No". |
|  | **Added value reward systems** | When the data indicates the added value of reward systems | **Respondent 1:** "Yes maybe yes. Yes, yes, yes, a bit of reward. It is a bit of appreciation for what you have done. Uh, we now have one of *** we did get a lot of appreciation because he really did uh encourage and support a lot and that was really nice. And uhm and you also saw that when he was here saying interim. Such a positive approach. And then we got lunch. It only has to be something small, sometimes an ice cream. And uh sometimes you also see, yes this is a bit different. But for example, with sick people. Uh well they might have been on sick leave for two years. Yes, uh you run yourself out of the snot for that person".  **Respondent 12:** "Yes, but appreciation, and I even notice this in my own group, in the group of managers, expressing appreciation uh really doesn't happen much, and we do try to do it in the department. And yet you often get bogged down in giving feedback, but not mentioning the extra positive and that remains difficult". |
|  | **Skills LM working** | When the data indicates what knowledge, skills and characteristics people need to work LM | **Respondent 5**: ‘’I think it's a difficult question. I don't think there's really an answer to that.’’  **Respondent 7**: ‘’Belt training in any case. But that really has to do with that it is important to make sure that you understand the underlying thought of LM, because it is not just a model, there is really a thought behind it. I think it's just really good to be taken into that and to go through that as well.’’  **Respondent 11**: ‘’I think you could actually do this at any level of education I think.’’  **Respondent 19**: ‘’Decisive. You have to have discipline. Perseverance.’’ |
| **Contribution LM to Strategic objectives** | **Contribution LM to strategic objectives** | When the data indicates the how the LM projects could contribute to the strategic objectives | **Respondent 18:** “And you may have vitality week, but it's mainly focused on the people who work here in the office in the A tower. But it's not for nurses. Because nurses what works here. They can't sign up for yoga classes at all, because they just have to take care of the patients. Their work can't wait. So, they always hit the nail on the head with that. And I think just more dissatisfaction towards the nursing staff. You always see, office staff is there, but the nurses are not” |
|  | **Contribution LM to personal development** | When the data indicates the contribution of LM to the employee’s personal development | **Respondent 15:** "By just making things smaller. Making notes with okay, I have to buy there and there, for Christmas I have to buy this, buy that. That you make notes so you make things smaller for yourself, so you get peace in your head, so a bit more space and maybe put things into perspective. But I'm not consciously working on it privately, not that I think "oh I'm going to put a Lean on that".  **Respondent 17:** "Well, I think I've said a lot of times, but looking at problems a bit differently. And yes, also because it is such a large organization. Not just. Sometimes you don't just need your department, you need other departments. And the best way to do that too is to just have it clear what your goal is, what a problem is and then be able to ask more focused questions. Yes, if you need them. Yes. Is that still then. |
|  | **Impact LM on quality Healthcare** | When the data indicates the impact from LM on the overall quality of healthcare | **Respondent 5:** "Well what I'm working on now is the discharge process. That should ultimately ensure that patients are discharged faster. So yes, that could then naturally lead to more admissions".  **Respondent 9:** "Yes, improving. All our Lean projects what we see, you do see improvement whether we are talking about pressure ulcers with new mattresses and a different way of scoring. Or we're talking about uh, there's a, I think you're talking about next week about dying phase where very nice measures have been made. That when someone is dying, it's there so that all that improves that quality". |
| **Alignment LM and Strategic Objectives** | **Alignment LM & strategy** | When the data indicates how there is alignment between strategic objectives and LM projects | **Respondent 16**: “A brief check takes place prior to the start of a LM project on the extent to which this improvement initiative contributes to one of the organization's strategic goals, so a strategic alignment check. Also, before allocating people, time, and budget for the LM project, a check takes place whether the LM project is in line with the organization's priorities. We periodically check the measurable goals during progress meetings.’’ |
|  | **Involvement in Strategic Objectives** | When the data indicates whether the employees feel involved in achieving the strategic objectives. | **Respondent 4:** "Really difficult, yes. Because I do enjoy coming to work, but I don't necessarily feel that *name organization* is doing a lot about it. Uhm, I think I just have a nice job because I have a nice team and really like the patient category. So, I don't know to what extent the board contributes to that".  **Respondent 6:** "Well not really one at all actually. I don't necessarily feel involved in when I think about what they do for a good work-life balance. Yeah, I don't really notice that much of that effectively. No actually". |
|  | **LM & organizational culture fit** | When the data indicates how LM fits in the organizational culture | **Respondent 17:** “Yes, I think in itself it is good because you also, yes you have to look at your problem from different angles. So, in that respect I do think in such a large organization it is very relevant to use it, but it is. Yes, if you really do it on a practical level. Yes, then I actually also think it's a good way because you learn to think very differently than just doing it instead of really getting into the rationale or the problem. So, I think lean fits within the culture”.  **Respondent 18:** "Yes, with this culture it fits well, I think. Yes fine. It gives you a handle to also focus on problems that are there, that can be improved". |
|  | **LM & organizational structure fit** | When the data indicates how LM fits in the corporate structure | **Respondent 19**: ‘’Yes, I think it does fit in well because it does give nice guidance. But yes, you do need time to work it out. We need to be able to hire more staff so that we also have time and space for improvements.’’  **Respondent 20**: ‘’Well, I don't know that very well because I'm not that much into the organization of course.’’ |
