# Supplementary material for: Alignment and contribution of lean management practices to strategic objectives in a healthcare context: a qualitative study in a university medical centre
Source: BMJ Open. 2025 Dec 15;15(12):e099758. doi: 10.1136/bmjopen-2025-099758 (PMC12706196; doi:10.1136/bmjopen-2025-099758)
Supplement: Supplementary file 2 [file bmjopen-2025-099758.reviewer_comments.pdf]

PEER REVIEW HISTORY

BMJ Open publishes all reviews undertaken for accepted manuscripts. Reviewers are asked to complete a checklist review form and are provided with free text boxes to elaborate on their assessment. These free text comments are reproduced below.

ARTICLE DETAILS

Title (Provisional)

Alignment and Contribution of Lean Management Practices to Strategic Objectives in a Healthcare Context: A Qualitative Study in a University Medical Centre

Authors

Zuidland, Anouk; Roemeling, Oskar; Aij, Kjeld H.; Pu, Lihui; Vijn, Femke

---

VERSION 1 - REVIEW

**Reviewer**	**1**
**Name**	**Abuzied, Yacoub**
**Affiliation**	**King Fahad Medical City, Nursing**
**Date**	**06-May-2025**
**COI**	**None**

I want to extend my gratitude for the opportunity to review this paper. It's a pleasure to engage with work that sheds light on a topic of such significance, particularly one that is seldom addressed in the existing literature. The paper contributes valuable insights to the field and opens up new avenues for discussion and exploration.

- The A abstract looks well-summarized and written.

- P4, please move the strengths and limitations from this page and keep it at the end of the discussion part.

- P5, under the introduction, the authors mentioned "Healthcare organizations are facing unpredictable challenges such as improving patient outcomes,

reducing healthcare costs, and providing high quality patient care". I suggest to add this clinical study to support your sentence with this citation as follow:

Abuzied Y, Maymani H, AlMatouq B, AlDosary O. Reducing the length of stay by enhancing the patient discharge process: using quality improvement tools to optimize hospital efficiency. Global Journal on Quality and Safety in Healthcare. 2021 Feb 1;4(1):44-9.

- The introduction in general is well-written and summarized, just please add the aim of the study to the end of the introduction.

- I prefer to to re-write this sentence of the research question: "The research question for this study was" to "The research question for this study is"

- Under the methods, move the aim of the study to be at the end of the introduction.

- Under the methods, please cite "Our study was guided by a conceptual lens, the ConModel." with a primary reference.

- The authors mentioned "the interviews were recorded and later transcribed to facilitate the coding process". Please explain more about the recording process and the transcription process like the language used, ... etc.

- Under the settings, please explain more about the interview setting and how did you insure the privacy.

- Under the data collection, the authors mentioned "aligning

with Guest et al. [20], who found saturation after nine interviews in their study." I don't see that its needed, you can delete it as the saturation is well defined as a saturation process.

- In Table 1, What is the use of having the Year of Birth, I suggest to replace it with the years of experience. Or take it out.

- In Table 2, the authors mentioned that "After the 18th interview,

no new insights emerged, indicating sufficient coverage of the study topics" But I can see in Table 2, 22 respondents. Please explain it.

- Please list the strengths of the study at the end of the discussion part.

- The rest of the sections looks good and well-written.

I hope that the tips help to improve the study and that it can be presented.

**Reviewer**	**2**
**Name**	**Lachman, Peter**
**Affiliation**	**Royal College of Physicians of Ireland, Quality**
**Date**	**18-Aug-2025**
**COI**	**None**

The paper is interesting and though it does not tell us much that is new it confirms that if there is a disconnect with strategy and action on the frontline then methods such as lean will not work.

My concern is that it is not clear whether the hospital has Lean as its strategic way of operation or whether it is a hospital, with strategic Lean is not the way it operates.

You could summarise the strategies and then link them to the projects.

If it is a Lean hospital then it has failed in the implementation of the strategy.

True Lean organisations like Virgina Mason or Theda Healthcare have Lean as their strategic way of thinking - i.e. it is their culture and they do not do Lean projects - Lean is the way they think and work at every level of the organisation. - everyone from executives and managers to front line staff use lean in all they do.

From your paper It seems yours is a hospital that does Lean projects and is not a Lean organisation.

Therefore there is nothing surprising in your results.

I recommend that you read the assessment from the work done in England with Virginia Mason on hospitals adopting Lean and you will see similar results.

https://www.wbs.ac.uk/news/six-key-lessons-from-the-nhs-and-the-virginia-mason-institute-partnership/

And there have been assessments on lean methodology application in England and Ireland e.g.

Burgess N, Radnor Z. Evaluating Lean in healthcare. Int J Health Care Qual Assur. 2013;26(3):220-35. doi: 10.1108/09526861311311418. PMID: 23729126.

https://academic.oup.com/intqhc/issue/31/Supplement_1

To summarise an interesting paper does not tell me more than I know.

What is valuable is the way your methods could be applied for a hospital to assess the connect between strategy and frontline action that could be a shorter case study. report but a paper on a method to assess the relationship between strategy and the frontline

---

**Reviewer**	**3**
**Name**	**Martin-Misener, Ruth**
**Affiliation**	**Dalhousie University**
**Date**	**01-Sep-2025**
**COI**	**None**

Thank you for the opportunity to review this interesting article.

1. It would be helpful to provide the rationale for why the setting was selected.

2. How did doing the site visits contribute to the study? How many people from the research team were involved in the visits? What data were collected during the site visits and how was it analyzed?

3. A brief paragraph telling readers about the positionality of the researcher(s) would be useful.

4. Table 1 contains information that taken together could make participants identifiable. I recommend that you summarize the data, for example, the total number of participants in each job title and the total number from each department. Year of birth is not relevant to the study and should be removed.

5. Line 36 on page 10, Results section. “This chapter…” would be better stated as “This section…)

6. Under results, on page 13 there is a sentence: “This indicates a misfit between the people and the structure, and between the people and the culture, which is typical for this case site.” Please elaborate/clarify the last phrase i.e. “which is typical for this case site” or consider removing it.

7. As the authors note, nurses in the project seem to have had a different experience of LEAN than other participants. This was evident to me as I read the quotes in the article. It would be useful to add a short paragraph or two talking about this along with a brief discussion in the context of relevant related literature.

VERSION 1 - AUTHOR RESPONSE

**Reviewer 1 comment**	**Author response**
**Section: Abstract**	
The abstract looks well-summarized and written.<br>- P4, please move the strengths and limitations from this page and keep it at the end of the discussion part.<br>- P5, under the introduction, the authors mentioned "Healthcare organizations are facing unpredictable challenges such as improving patient outcomes,<br>reducing healthcare costs, and providing high quality patient care". I suggest to add this clinical study to support your sentence with this citation as follow: Abuzied Y, Maymani H, AlMatouq B, AlDosary O. Reducing the length of stay by enhancing the patient discharge process: using quality improvement tools to optimize hospital efficiency. Global Journal on Quality and Safety in Healthcare. 2021 Feb 1;4(1):44-9.	Thank you for your comments.<br><br>Regarding the limitations, we follow the BMJ Open proposed structure which requires us to place the Strength and Limitations after the structured abstract. However, we do have a separate Limitations section that is part of the discussion section of our manuscript.<br><br>The provided source (Abuzied, et al.) helps us to further substantiate our claim on challenges in healthcare. We have added the citation to our manuscript where we explain issues with performance, such as poor patient flow or inadequate resource utilization.

**Section: Introduction**	
- The introduction in general is well-written and summarized, just please add the aim of the study to the end of the introduction.<br>- I prefer to to re-write this sentence of the research question: "The research question for this study was" to "The research question for this study is"	Thank you for your suggestion, we have adjusted our research question following your advice. Moreover, as mentioned before, we have introduced the citation into our main manuscript, and we now end our introduction by sharing the main aim of our research.
**Section: Methods**	
- Under the methods, move the aim of the study to be at the end of the introduction.<br>- Under the methods, please cite "Our study was guided by a conceptual lens, the ConModel." with a primary reference.	Thank you for your suggestion, here we have taken your advice and we moved the aim of the study towards the end of the introduction section.<br><br>In addition, we have added references to the Congruence Model (ConModel) in the Methods section. We now cite:<br><br>Nadler DA, Tushman ML. A model for diagnosing organizational behavior. Organ Dyn. 1980;8:35-51.<br><br>Kloevekorn L, Roemeling O, Fakha A, Hage E, Smailhodzic E. Decarbonizing surgical care: a qualitative systematic review guided by the Congruence Model. BMC Health Serv Res. 2024;24(1):1456.
- The authors mentioned "the interviews were recorded and later transcribed to facilitate the coding process". Please explain more about the recording process and the transcription process like the language used, ... etc.	During the interviews, we used multiple ways of recording the audio: smartphone and / or laptop. All interviews were audio-recorded with participants' consent and transcribed verbatim (i.e., including false starts) to preserve the

	authenticity of responses. Transcripts were not edited for grammar or clarity to ensure accuracy in qualitative analysis. We have updated this section in the manuscript to clarify our approach.
- Under the settings, please explain more about the interview setting and how did you insure the privacy.	Interviews were conducted exclusively with participants who provided voluntary, informed consent. Sessions took place in private, undisturbed settings (e.g., closed offices or reserved meeting rooms) to ensure confidentiality. All audio recordings have been deleted after completing the transcripts and transcripts remain securely stored, accessible only to the research team, and have not been shared with the case site or external parties. We have updated the methods to explain this more clearly, and we have included our changes in the section on Data collection.
- Under the data collection, the authors mentioned "aligning<br>with Guest et al. [20], who found saturation after nine interviews in their study." I don't see that its needed, you can delete it as the saturation is well defined as a saturation process.	Thank you for this suggestion, and we agree that saturation is a generally well understood term in qualitative studies (making this citation less relevant). We have removed it from the main text.
- In Table 1, What is the use of having the Year of Birth, I suggest to replace it with the years of experience. Or take it out.<br>- In Table 2, the authors mentioned that "After the 18th interview,<br>no new insights emerged, indicating sufficient coverage of the study topics" But I can see in Table 2, 22 respondents. Please explain it.<br>- Please list the strengths of the study at the end of the discussion part.<br>- The rest of the sections looks good and well-	Thank you for these suggestions. We followed the broader shared advice from multiple reviewers and the editorial office and we removed the date of birth from our Table 1 on the participants. Moreover, we restructured Table 1 to be more concise, in line with comments of R3.<br><br>In relation to Table 2, we aimed to inform the reader that after the 18th interview we seemed to have reached saturation. The total number of conducted interviews is 22, but after the 18th

written.	interview no new insights evolved from the additional participants. We realize now that our wording could lead to confusion and we slightly adjusted the text to better explain this to our readers.
I hope that the tips help to improve the study and that it can be presented.	We would like to take this opportunity and thank you for taking the time to read through our manuscript and provide us with these useful comments.
**Reviewer 2 comment**	**Author response**
The paper is interesting and though it does not tell us much that is new it confirms that if there is a disconnect with strategy and action on the frontline then methods such as lean will not work.<br><br>My concern is that it is not clear whether the hospital has Lean as its strategic way of operation or whether it is a hospital, with strategic Lean is not the way it operates.<br>You could summarise the strategies and then link them to the projects. If it is a Lean hospital then it has failed in the implementation of the strategy.<br><br>True Lean organisations like Virgina Mason or Theda Healthcare have Lean as their strategic way of thinking - i.e. it is their culture and they do not do Lean projects - Lean is the way they think and work at every level of the organisation. - everyone from executives and managers to front line staff use lean in all they do.<br><br>From your paper it seems yours is a hospital that does Lean projects and is not a Lean organisation. Therefore there is nothing	We acknowledge your observation that our study confirms existing knowledge about the challenges of Lean implementation when there is a disconnect between strategy and frontline action. However, we believe our work contributes to the literature in two key ways:<br><br>Contextual Nuance: Our qualitative study provides a detailed, empirical exploration of how this disconnect manifests in a specific healthcare setting, which may offer practical insights for other organizations facing similar challenges.<br><br>Methodological Contribution: As you noted, our approach to assessing the alignment between strategy and frontline action could serve as a useful framework for other hospitals or institutions. As the application of the Congruence Model is not that common in the healthcare domain.<br><br>It is difficult to reflect on the research site in terms of it being a true Lean organization. What we can share is that the director, responsible for

surprising in your results.<br><br>I recommend that you read the assessment from the work done in England with Virginia Mason on hospitals adopting Lean and you will see similar results.<br><br>https://www.wbs.ac.uk/news/six-key-lessons-from-the-nhs-and-the-virginia-mason-institute-partnership/<br><br>And there have been assessments on lean methodology application in England and Ireland e.g.<br>Burgess N, Radnor Z. Evaluating Lean in healthcare. Int J Health Care Qual Assur. 2013;26(3):220-35. doi: 10.1108/09526861311311418. PMID: 23729126. https://academic.oup.com/intqhc/issue/31/Supplement_1	all departments, has a PhD in the lean management domain - and tries to actively engage and apply lean methodologies. Moreover, staff is being trained and informed on lean principles, and there is an ongoing effort to make process improvements. Perhaps our study does reflect the challenges of aiming for lean practices, in a highly complex environment.<br><br>We thank you for your suggested readings, and we have added the study of Burgess and Radnor to our discussion section in order to underline that we add to our current understanding.
To summarise an interesting paper does not tell me more than I know. What is valuable is the way your methods could be applied for a hospital to assess the connect between strategy and frontline action that could be a shorter case study. report but a paper on a method to assess the relationship between strategy and the frontline	Thank you for your review and suggestions, we have updated our future research section to highlight that our applied methods (applying the congruence model) could be used to assess connections between strategy and frontline action.
**Reviewer 3 comment**	**Author response**
**Section: Methods**	
It would be helpful to provide the rationale for why the setting was selected.	Thank you for this comment. Following your advice, we have added more background on why

	we selected the university medical center for our studies under the Setting section. Our rationale mainly relates to the experience with LM that is present within our studied setting. With well over 10 years of LM practice experience and LM in-house training, we were confident that we could discuss LM with well-informed professionals.
How did doing the site visits contribute to the study? How many people from the research team were involved in the visits? What data were collected during the site visits and how was it analyzed?	The site visits enabled researchers AZ and FV to familiarize themselves with the research environment. These visits provided contextual insights that enhanced our interpretation of interview statements. While conducting interviews and taking research notes during the visits, we did not include the site visits themselves in the formal analysis. However, they played a critical role in clarifying and contextualizing the interview data. We now realize that adding the site visits as part of the data table might lead to confusion. We have removed the sites visits from Table 2 and now mention the visits in the main text.
A brief paragraph telling readers about the positionality of the researcher(s) would be useful.	Thank you for this suggestion and we agree that sharing some background on the research team is helpful. We have added a paragraph on positionality at the beginning of the methods section where we shortly describe the research team members. This section reads as follows:<br>“In order to highlight any biases or preconceptions that might exist in the research team, we provide the positionality of the team members in this paragraph. The main researchers in this study (OR, KA) have significant experience with research aimed at LM in healthcare. In addition, OR holds a managerial

	position in education, and KA holds a position of director in a healthcare setting. As a result, they are familiar with issues that arise when managing a complex environment, which informs their reflections of observations in the field. Researchers AZ and FV, both holding a bachelor's degree (BSc) at the time, were completing their master's degree (MSc) during this study, and both have a background in business studies. Both AZ and FV had limited exposure prior to this research to the managerial side of the healthcare context. Senior researcher LP has a background in nursing education, practice and research, who has rich experience in qualitative research methods in healthcare."
Table 1 contains information that taken together could make participants identifiable. I recommend that you summarize the data, for example, the total number of participants in each job title and the total number from each department. Year of birth is not relevant to the study and should be removed.	Thank you for your suggestions for our Table 1. We followed your advice and have restructured Table 1. We choose to not combine job titles as this would make it more difficult to link quotations to interviewee IDs. However, if required, we are obviously willing to make some further changes to Table 1.
**Section: Results**	
Line 36 on page 10, Results section. "This chapter…" would be better stated as "This section…)	We agree with your suggestion and we have changed the text following your advice.
**Section: Discussion**	
Under results, on page 13 there is a sentence: "This indicates a misfit between the people and the structure, and between the people and the culture, which is typical for this case site." Please elaborate/clarify the last phrase i.e. "which is typical for this case site" or consider	It seems that the part of the sentence: "which is typical (...) site" is a leftover from an earlier sentence or statement. We regret that we missed this during the final cleanup of the original manuscript and we thank you for bringing this to our attention. We have removed this part of the

removing it.	sentence from the revised manuscript.
As the authors note, nurses in the project seem to have had a different experience of LEAN than other participants. This was evident to me as I read the quotes in the article. It would be useful to add a short paragraph or two talking about this along with a brief discussion in the context of relevant related literature.	Thank you for highlighting this point. We added a short paragraph to discuss the different experiences of nurses and staff with leader or management responsibilities, and provided some suggestions with related literature.

VERSION 2 - REVIEW

Reviewer 2

Name Lachman, Peter

Affiliation Royal College of Physicians of Ireland, Quality

Date 17-Oct-2025

COI

Thank you for addressing my recommendations.

VERSION 2 - AUTHOR RESPONSE

**Reviewer comments**<br>Reviewer: 2<br>Dr. Peter Lachman, Royal College of Physicians of Ireland<br>Comments to the Author:<br>Thank you for addressing my recommendations.<br><br>Reviewer: 2<br>If you have selected 'Yes' above, please provide details of any competing interests.: not applicable
**Author response**<br>We thank you again for your suggestions and we're happy to see that we were able to address your recommendations.
